# Genome-Wide Identification of R2R3-MYB Gene Family in Strawberry (*Fragaria vesca* L.) and Functional Characterization of *FvMYB103* in Cold Stress

**DOI:** 10.3390/ijms27020771

**Published:** 2026-01-13

**Authors:** Changjia Zhao, Zhe Chen, Wenhui Li, Deguo Han, Xiang Chen, Fenghua Huang, Lihua Zhang, Wanda Liu, Yu Wang, Xingguo Li

**Affiliations:** 1 Key Laboratory of Biology and Genetic Improvement of Horticultural Crops (Northeast Region), Ministry of Agriculture and Rural Affairs, National–Local Joint Engineering Research Center for Development and Utilization of Small Fruits in Cold Regions, College of Horticulture & Landscape Architecture, Northeast Agricultural University, Harbin 150030, China; z772583jia@163.com (C.Z.); zhechen0313@163.com (Z.C.); wenhuili@neau.edu.cn (W.L.); deguohan@neau.edu.cn (D.H.); cx01160202@163.com (X.C.); zlh15009205209@163.com (L.Z.); 2 Institute of Agricultural Remote Sensing and Information of Heilongjiang Academy of Agricultural Sciences, Harbin 150030, China; hfh71@126.com (F.H.); liuwanda@haas.cn (W.L.); haaslwd@126.com (Y.W.)

**Keywords:** cold tolerance, *Fragaria vesca* L., R2R3-MYB transcription factor, abiotic stress

## Abstract

*Fragaria vesca* L., a widely distributed model species, serves as a key resource for studying the evolution and genetics of the Fragaria genus. Research has shown that R2R3-MYB transcription factors are crucial for plant growth and development. However, their specific role in cold resistance in *F. vesca* is not well understood. In this study, we used the latest genome data for the strawberry (*F. vesca* v6.0). We performed a genome-wide identification of the R2R3-MYB gene family in *F. vesca*. We identified a total of 106 R2R3-FvMYBs. Based on their predicted functions in plants, we classified these genes into 25 distinct subfamilies. We then conducted a comprehensive bioinformatics analysis of this family. We performed a detailed examination of the R2R3-FvMYBs structures and physicochemical properties. This analysis provided five key parameters for each protein: molecular weight, the number of amino acids, theoretical isoelectric point, grand average of hydropathicity (GRAVY), and instability index. Gene duplication analysis suggested that segmental duplications were a primary driver of the proliferation of this gene family. Promoter cis-acting element prediction revealed that a large proportion of R2R3-FvMYBs possess elements predominantly associated with phytohormone responsiveness and biotic/abiotic stress responses. Quantitative real-time reverse transcription PCR (qRT-PCR) results confirmed that the expression levels of several R2R3-FvMYBs were upregulated under cold stress. Furthermore, compared to wild-type controls, the overexpression of *FvMYB103* in *Arabidopsis thaliana* enhanced cold tolerance, accompanied by increases in the relevant physiological indices. Collectively, these findings support further investigation into R2R3-MYB gene family to directly assess their contribution to cold resistance.

## 1. Introduction

As one of the abiotic stress factors, cold stress significantly impacts plant growth and can lead to crop yield reduction or even plant death in severe cases. Plants have evolved adaptive mechanisms through long-term evolution to respond to cold stress, enabling them to rapidly perceive stress signals and enhance their adaptability at both the physiological and molecular levels [1]. Cold injuries, encompassing both freezing and chilling damage, can cause irreversible harm to plant growth and development [2]. Since relying solely on their innate capacity is often insufficient to effectively withstand low-temperature stress, researchers have introduced cold tolerance genes into plants to enhance their hardiness, thereby cultivating new varieties with improved cold resistance [3,4].

*Fragaria vesca* L. is a diploid plant of significant research value, characterized by its relatively small genome, short life cycle, and stable genetic traits. Due to the high sequence similarity between diploid and octoploid strawberries, it is frequently utilized as a model plant for studying berry species [5]. In recent years, the release of updated genomic data for *F. vesca* has provided more robust support for research and breeding efforts in strawberry and other rosaceae species. It has been confirmed that *Fragaria vesca* L. can maintain stable cell activity and physiological metabolic levels under cold environments, and its stolon tissues can maintain a high survival rate after 72 h of cold stress at −5 °C, and have a typical cold resistance-related phenotype. However, the R2R3-MYB gene family, a key regulator of plant abiotic stress responses, still has few reports in regulating cold stress.

The mechanisms underlying cold and drought tolerance in strawberries are a major research focus. In recent years, studies on the cold resistance of *Fragaria vesca* L. have been conducted from physiological, transcriptomic, and molecular biological perspectives. From a physiological standpoint, the present study investigated the effect of nitric oxide donor-functionalized sodium alginate coating on maintaining the quality and antioxidant enzyme activity of strawberries after cold storage [6]. The research demonstrated that SA + SNP coating presents a promising method for reducing microbial decay and preserving strawberry storage quality [7]. The study isolated the *FvICE1* gene from *Fragaria vesca* ‘Hawaii 4’. Research using molecular biology has shown that overexpressing the *FvICE1* gene can improve strawberry plant’s tolerance to both cold and drought [8]. In a related study on peaches, it was reported that two transcription factors (TFs), PpERF4 and PpERF061, both regulate the response to cold stress. However, they function differently. PpERF4 directly activates the genes PpCBF1 and PpVIN2. In contrast, PpERF061 acts as a broader repressor for related genes. A notable finding is that cold stress triggers PpERF061 to suppress PpCBF1 expression. This suppression then allows PpCBF1 to begin positively regulating PpVIN2. This series of events ultimately reduces cold tolerance in the peach [9]. Studies have demonstrated that several TFs and functional genes are essential for how plants respond to abiotic stress, including WRKY and bZIP transcription factors [10,11,12,13,14]. MYB TFs are particularly significant and the most extensively studied classes of plant transcription factor [15].

MYB TFs can be classified into three categories, based on their domain structures [16]. R2R3-MYB TF is the most abundant type in plants, which not only features a characteristic two-domain architecture but also modulates the critical processes of plant growth, secondary metabolism, phytohormone signaling, and abiotic stress responses [17,18,19]. In response to diverse abiotic stresses, plant R2R3-MYB genes are upregulated and orchestrate adaptive responses to cold, drought, and ABA signaling [20]. Studies have shown that the expression of the R2R3-MYB family member SpMYB1 increased under drought and cold treatments. It was found that in SpMYB1-overexpressing lines, the expression of antioxidant and stress-related genes (e.g., *SlDREB2, SlABF4, SlCAT, SlPOD, SlSOD, SlCAM, SlFLW10, SlRubisco, SlMDH, SlGlut, SlABF2, SlABF3*) was significantly enhanced [21]. Research discovered that CaMYB306 impairs cold tolerance in pepper by suppressing calcineurin B-like interacting protein kinase gene 13 (CaCIPK13) transcriptional activity, which modulates the reactive oxygen species system and the expression of cold-related genes [22]. There is evidence that the PpcMYB20 is significantly influenced by abiotic stress and negatively regulates salt and cold tolerance in pears [23]. A study reported that VaMYB4a enhances cold tolerance in grapevines by physiologically mediating osmoregulation, ROS scavenging, and stomatal movement, and molecularly by directly upregulating CBF and COR genes [24]. A researcher found that *Pyrus hopeiensis* flowers PhMYB62, belonging to the R2R3-MYB subfamily, play a positive role in cold stress response [25]. All in all, these findings show that R2R3-MYBs have a variety of regulatory effects on plant cold tolerance, but their effect on the functional characteristics of *F. vesca* are still difficult to determine.

In the present study, we identified the R2R3-MYB gene family member *FvMYB103* through comprehensive genome-wide identification. Then, the heterologous overexpression of *FvMYB103* was functionally characterized in *Arabidopsis thaliana*, and its regulatory role in mediating plant cold stress was verified. This research lays a foundation for elucidating the R2R3-MYB gene family’s functions and serves as a valuable resource for molecular breeding aimed at enhancing freezing tolerance in *F*. *vesca*.

## 2. Results

### 2.1. Genome-Wide Identification and Phylogenetic Analysis of R2R3-MYB Gene Family in Fragaria vesca

**Figure 1:**
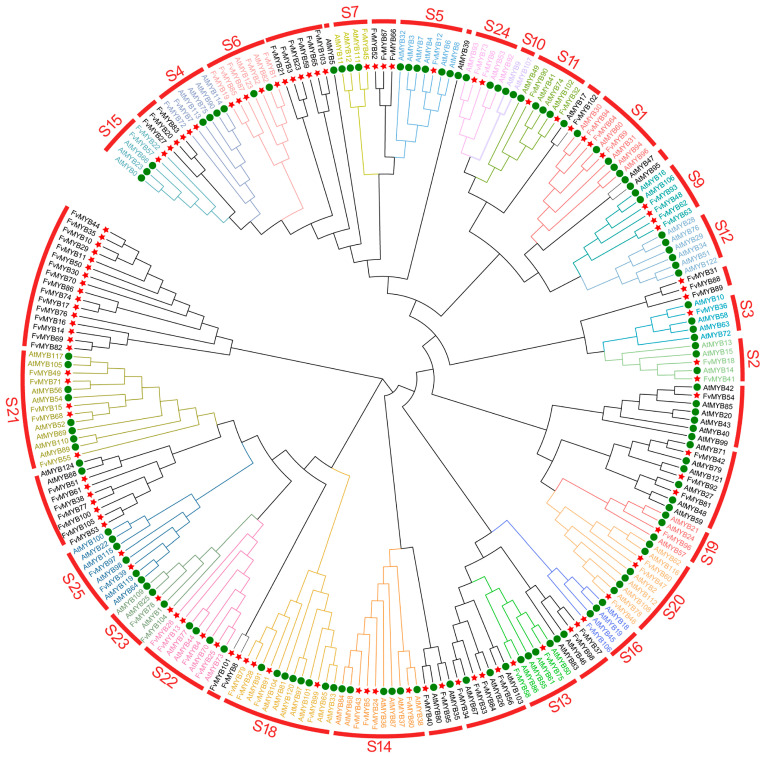
Phylogenetic analysis of R2R3-MYB gene families from *Arabidopsis thaliana* and strawberry (*F. vesca*). The colored areas represent distinct functionally classified subfamilies of R2R3-MYB gene family, as shown in Figure 1. Each subfamily is indicated by a different color; “Fv” denotes strawberry (*Fragaria vesca* L.), and “At” denotes *Arabidopsis thaliana* (L.) *Heynh*. The green circle represents the gene in Arabidopsis, and the red five-pointed star represents the gene of *F. vesca*.

A HMM (Hidden Markov Model) search for the MYB domain (PF00249) was performed based on the latest *Arabidopsis thaliana* V10.2 and its genomic data. After domain screening, 124 *Arabidopsis* R2R3-MYB genes and 106 *Fragaria vesca* R2R3-MYB genes were identified. The classification of MYB proteins is based on the count of their adjacent repeat sequences, namely R1, R2R3, R3, and R4 repeats. Most MYB genes in plants encode R2R3-MYB proteins. This study classified the R2R3-MYB gene families of *Arabidopsis thaliana* and *F. vesca* into 25 subfamilies, following the phylogenetic and functional analysis of R2R3-MYBs in *Arabidopsis*. These subfamilies exhibited two classification patterns: conserved subfamilies with a balanced number of genes between *Arabidopsis* and *F. vesca* (S1, S3, S5, S8), and biased subfamilies dominated by the gene count of one species (S2, S4, S6, S7, S9, S10). The largest subfamily was S8, containing 58 genes (32 from *Arabidopsis* and 26 from *F*. *vesca*), accounting for 26.9% of the total MYB gene family. S10 was the smallest subfamily, with only 11 genes (7 from *Arabidopsis* and 4 from *F. vesca*). Notably, the S7 subfamily comprised 24 genes, with *F. vesca* enriched with 21 of them; the S4 subfamily contained 23 genes, including 19 from *Arabidopsis* (as shown in Figure 1).

### 2.2. Analysis of the Gene Structures and Physicochemical Properties of the R2R3-MYB Gene Family

Through conserved motif analysis, we characterized five physicochemical parameters (amino acid count, molecular weight, theoretical pI (isoelectric point), instability index (II), and GRAVY) of the 106 R2R3-FvMYB proteins identified in *F*. *vesca* (Statistical analyses of the data are provided in Table S1). The number of amino acids forms the basis of the protein’s structural complexity and directly determines its molecular weight [26]. The number of amino acids in R2R3-FvMYB proteins exhibited considerable variation, ranging from a minimum of 188 aa in FvMYB66 to a maximum of 655 aa in FvMYB16, resulting in a difference of 467 aa. From a functional perspective, 75% of R2R3-FvMYB proteins possessed an amino acid count concentrated within the 220–400 aa range, which aligns with the typical structural feature of R2R3-MYB TFs that primarily bind DNA via their R2R3 domains to participate in transcriptional regulation. We considered that proteins that may participate in more complex regulatory networks with over 500 amino acids likely contain extra functional domains. Differences in amino acid length directly affect other characteristics. The molecular weight of R2R3-FvMYB proteins was closely correlated with their amino acid count, ranging from 21,409.53 Da for FvMYB66 to 74,722.99 Da for FvMYB16. The isoelectric point (pI) indicates its net charge at a standard physiological pH, which directly affects how the protein interacts with other negatively charged molecules and proteins [27]. The pI values for R2R3-FvMYB proteins ranged from 4.98 to 9.54, and the average pI was 6.72 ± 1.24. Within these proteins, 61 proteins (58.7%) were acidic and 43 proteins (41.3%) were basic, and we did not identify any neutral proteins. The instability index predicts whether a protein is stable in vitro, with an index below 40 suggesting stability and above 40 indicating instability [28]. The index for R2R3-FvMYB proteins ranged from 36.96 to 73.03, with an average of 53.2 ± 8.7. Only 12 proteins (11.5%) were classified as stable and the majority of 92 proteins (88.5%) were unstable. The amino acid count of stable proteins is mainly concentrated between 220 and 300 aa, and the pI value tends to be neutral or slightly acidic. These proteins may be involved in the basic life activities of plants and require long-term stability to maintain homeostasis. The grand average of hydrophilicity (GRAVY) reflects the overall hydrophilicity or hydrophobicity of protein, whose value is directly related to subcellular localization. Nuclear localization proteins are usually hydrophilic, while membrane localization proteins are usually hydrophobic [29]. R2R3-FvMYB proteins had negative GRAVY values, with an average of −0.72 ± 0.13, indicating that the FvMYB family consists of hydrophilic proteins. However, they were further categorized into 15 weakly hydrophilic proteins (14.4%) and 32 strongly hydrophilic proteins (30.8%). Based on GRAVY values, it can be inferred that the majority of R2R3-FvMYB proteins are likely localized to nucleus.

### 2.3. Chromosomal Localization of R2R3-MYB Gene Family

**Figure 2:**
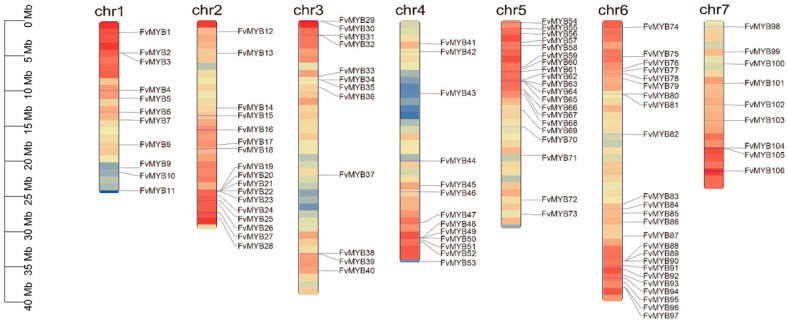
Chromosomal distribution of R2R3-MYB gene family in strawberry (*Fragaria vesca*). The color gradient of chromosomal segments reflects the spatial distribution inhomogeneity of genes, with darker colors representing higher gene density and lighter colors indicating lower gene density.

This study assigned systematic names to R2R3-FvMYB family members according to their order on the chromosomes (Renamed gene names are provided in Table S2). An analysis of the chromosomal locations of R2R3-FvMYB gene family members in *F. vesca* was performed. Figure 2 illustrates that the distribution of the 106 genes across the seven chromosomes is uneven. A higher number of genes were located on Chr2 and Chr6, whereas relatively fewer FvMYBs were found on Chr4. A discernible clustering phenomenon of R2R3-FvMYBs was observed on chromosomes such as Chr2, Chr5, and Chr6. By examining the specific positions of R2R3-FvMYBs on the chromosomes, it can be observed that genes located in different regions may correspond to distinct functional domains.

**Figure 3:**
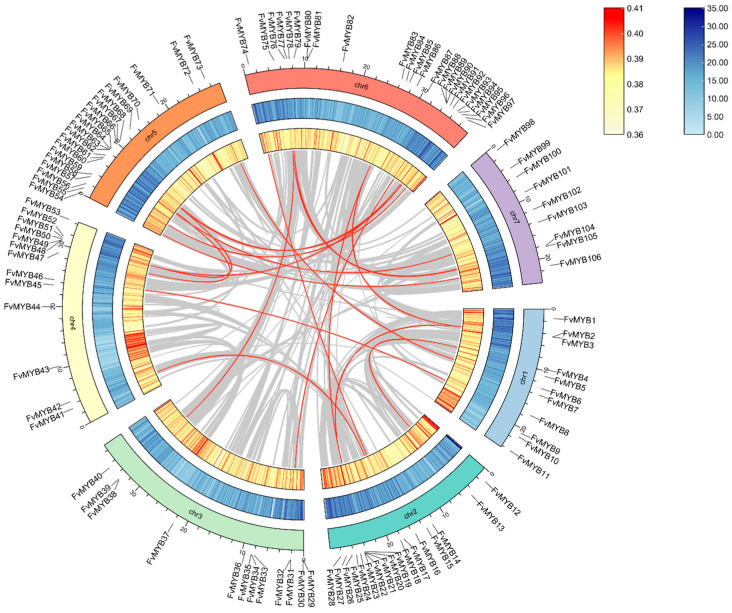
Species synteny and duplicated gene pair analysis in strawberry (*F. vesca*). In the figure, the red lines represent duplicated gene pairs, the gray areas denote syntenic blocks, and the surrounding colored blocks correspond to different chromosomes.

**Figure 4:**
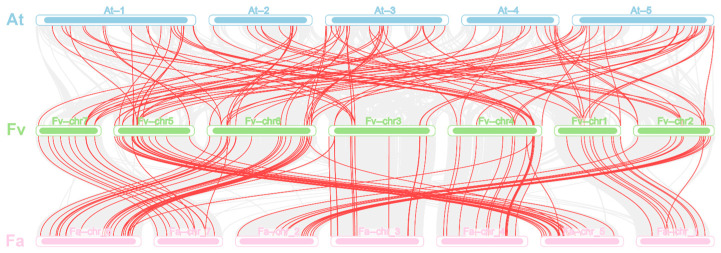
Interspecific synteny analysis among *Fragaria vesca*, *F. ananassa*, and *Arabidopsis thaliana*. Red lines indicate syntenic gene pairs, while the light gray areas represent syntenic blocks between the genomes of the different species.

We identified a substantial number of linked gene pairs, or syntenic connections, between different chromosomes in the *F. vesca* genome, indicating that segmental duplications have occurred widely throughout the genome. Multiple connections like the FvMYB19-FvMYB28 gene cluster on chromosome 2 and FvMYB87-FvMYB97 gene cluster on chromosome 6 were observed. The gene family commonly expands through genomic duplication events which fall into three main categories: tandem duplication, whole-genome duplication (WGD), and segmental duplication. The results of the synteny analysis for the *F. vesca* R2R3-MYB gene family are shown in Figure 3. The Circos plot illustrates the chromosomal distribution of R2R3-FvMYBs and visually confirms widespread duplications. The R2R3-MYB gene family exhibited a non-uniform distribution across chromosomes, with gene clusters resulting from tandem duplications present on specific chromosomes such as chr2 and chr6. Furthermore, MCScanX scan for syntenic blocks identified 21,293 duplicated gene pairs in the *F. vesca*–*F. ananassa* comparison; the *F. vesca*–*A. thaliana* comparison yielded 12,211 pairs from interspecific synteny analysis among *Fragaria vesca*, *F. ananassa*, and *Arabidopsis thaliana*. Among these three species, 106 members of the R2R3-MYB gene family exhibited syntenic relationships (as shown in Figure 4).

### 2.4. Analysis of Cis-Acting Elements in the Promoter Regions of R2R3-MYB Genes

**Figure 5:**
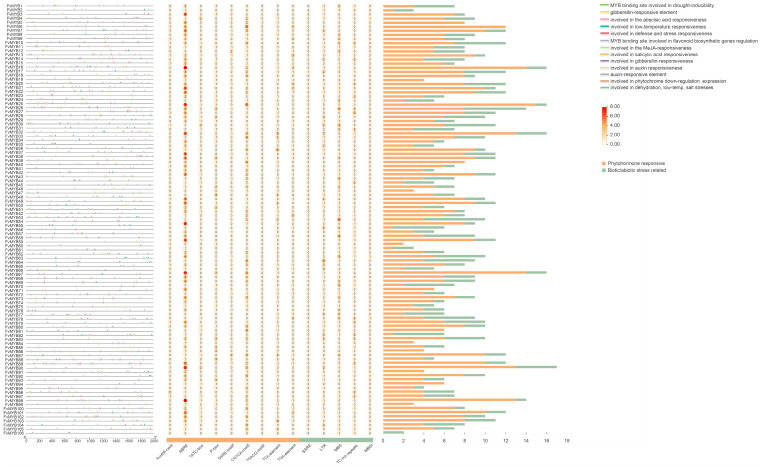
Cis-acting elements within the 2 kb sequences upstream of the start codon (ATG) of R2R3-FvMYB gene family members were predicted using PlantCARE; the vertical axis represents gene name, and the horizontal axis represents the main cis-acting elements, as shown in Figure 5.

Cis-acting elements within 2 kb sequences upstream of the start codon (ATG) of R2R3-FvMYB gene family members were predicted using PlantCARE (http://bioinformatics.psb.ugent.be/webtools/plantcare/html/, accessed on 10 May 2025), as shown in Figure 5. A total of 1790 cis-acting elements were predicted in the promoter regions of the 106 R2R3-FvMYBs. Most R2R3-MYBs featured promoter cis-element profiles dominated by motifs involved in phytohormone signaling and stress adaptation. Among these, the phytohormone-responsive elements mainly included those for auxin, ABA, MeJA, gibberellin, and salicylic acid. The elements related to biotic/abiotic stress responses were predominantly those involved in cold and drought stress. A small proportion of the elements were associated with flavonoid biosynthesis.

### 2.5. Expression Profiles of R2R3-FvMYBs in the Low-Temperature Transcriptome

**Figure 6:**
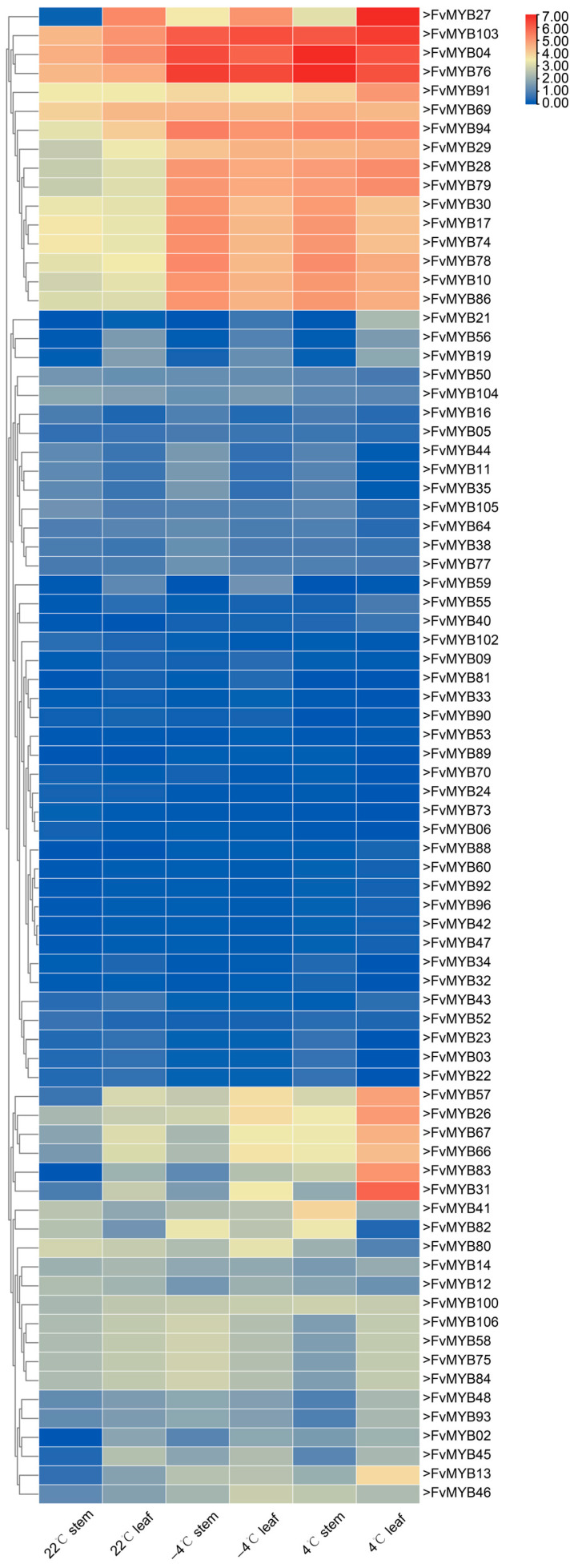
Expression profiles of R2R3-FvMYB gene family in strawberry leaves and stems following treatments at room temperature (22 °C), low temperature (4 °C), and freezing temperature (−4 °C). Figure 6 presents a heatmap of R2R3-FvMYB subfamilies, with the data subjected to cluster analysis. The heatmap was generated based on the RNA-seq expression data (FPKM ≥ 1) of R2R3-FvMYBs, which were log_2_-transformed. The color gradient from blue to red represents expression levels from high to low.

Building upon the analysis of *cis*-acting elements in R2R3-FvMYBs, the transcriptomic data of these genes under low-temperature conditions were further examined to study the expression patterns of R2R3-MYBs under low-temperature stress. *F. vesca* plants were exposed to two low-temperature treatments: chilling at 4 °C for 8 h and freezing at −4 °C for 8 h, and cultured at 22 °C as a control group. RNA sequencing (RNA-seq) results corresponding to R2R3-FvMYBs were extracted from the transcriptomic data, and clustering methods were employed for data analysis. After constructing RNA sequencing libraries and high-throughput sequencing, the expression maps of R2R3-FvMYBs were extracted from the transcriptome dataset, and traditional cluster analysis was used for data mining. Compared to the control (22 °C), the expression of 16 R2R3-FvMYBs was significantly altered following cold treatment, while the remaining 90 R2R3-FvMYBs showed no significant difference in expression level compared to the control. Subsequently, to verify the reliability of the RNA-seq data, we performed a validation experiment by quantitative real-time PCR (qRT-PCR): *F. vesca* plants were treated under the same cold stress conditions described above, and the stem and leaf tissue were collected identically, and specific primers were designed for four out of the sixteen significantly altered genes to detect their expression levels via qRT-PCR. As shown in Figure 6, cold treatment induced the expression of all four genes relative to controls, thereby providing direct evidence for their positive regulatory role in the cold stress response of *F. vesca*.

**Figure 7:**
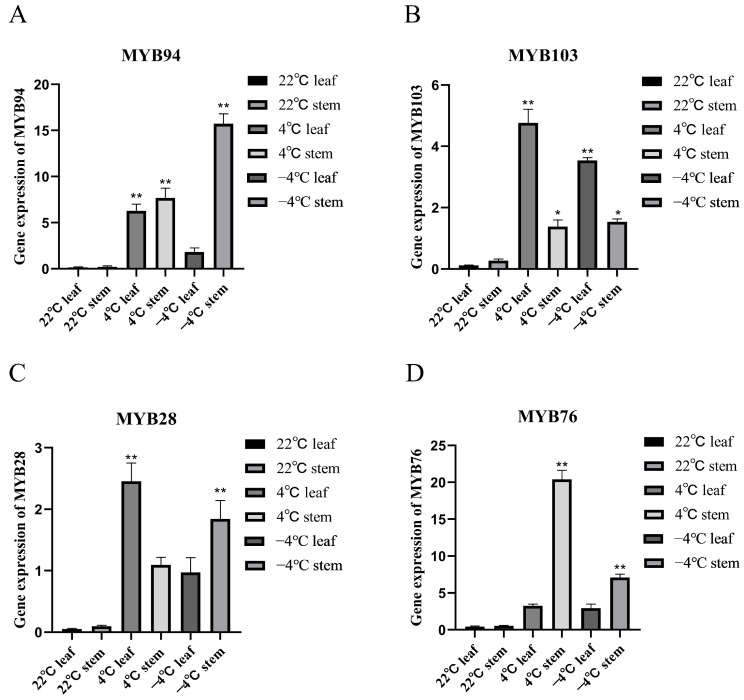
qRT-PCR validation of four selected R2R3-FvMYBs (**A**–**D**) in strawberry stems and leaves at 22 °C, 4 °C, and −4 °C confirmed their differential expression, with data processed using the 2^−ΔΔCt^ method. Data are expressed as mean ± SE of three biological replicates, with differences assessed by Student’s *t*-test. Differences were considered highly significant at *p* < 0.01 (**) and significant at *p* < 0.05 (*).

In this study, by treating *F. vesca* and subsequently measuring the expression levels of related genes, we found that *FvMYB103* was significantly activated following treatment at both 4 °C and −4 °C, with its expression markedly increased in roots and stems (as is shown in Figure 7). This indicates a strong response of *FvMYB103* to cold stress. This result is consistent with the trends observed in the transcriptome data.

### 2.6. Overexpression of FvMYB103 Gene in Arabidopsis thaliana

**Figure 8:**
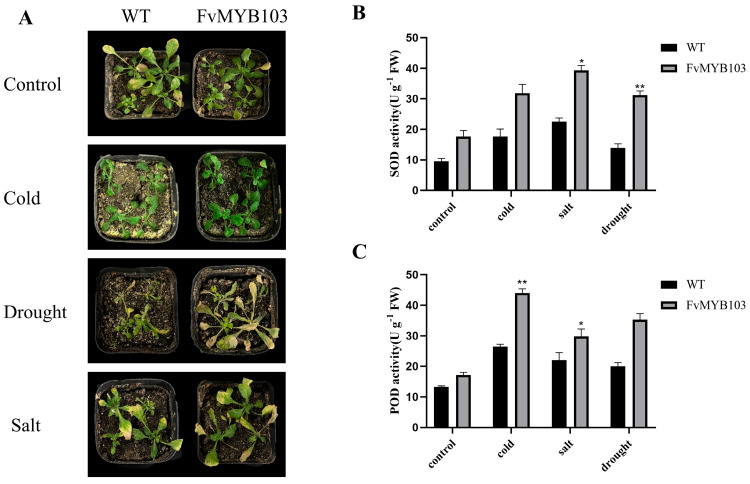
Phenotypes and antioxidant enzyme activities in wild-type and transgenic FvMYB103 *Arabidopsis* under three stress conditions. Plants were subjected to cold stress (−4 °C for 10 h), salt stress (irrigation with 200 mM NaCl for 5 d), or drought stress (water withholding for 5 d) (**A**). Activities of SOD and POD were then measured across all treatments (**B**,**C**). Significant differences were determined using one-way analysis of variance (ANOVA). Differences were considered highly significant at *p* < 0.01 (**) and significant at *p* < 0.05 (*).

We generated transgenic *Arabidopsis thaliana* plants overexpressing FvMYB103 using the Agrobacterium-mediated floral dip method. Genomic DNA was extracted for PCR verification, and lines exhibiting higher transgene expression were selected for subsequent low-temperature, drought, and salt stress treatments. Wild-type and transgenic *Arabidopsis* plants, which grow under normal growing conditions, were subjected to phenotypic observation and antioxidant enzyme assays under various stress conditions to assess their physiological responses. Under normal growth conditions, no significant differences were observed between the two. However, following low-temperature treatment, wild-type *Arabidopsis* leaves showed more pronounced water-soaking and wilting whereas the leaves of transgenic plants showed minimal water-soaking, less severe wilting, and overall milder damage. Further stress tolerance testing showed that transgenic *Arabidopsis* plants showed a much higher tolerance to all these stresses, tested under their ability to withstand cold, drought, and salt stress. Their performance is documented in Figure 8A; the plant’s ability to resist stress depends heavily on its antioxidant enzymes. These enzymes, essential for a plant’s healthy growth, development, and stress tolerance, manage harmful molecules that accumulate under difficult conditions [30]. In order to explore the physiological mechanism of FvMYB103, we measured two key antioxidant enzymes and compared peroxidase (POD) and superoxide dismutase (SOD) activity in both normal and transgenic plants. The results showed that the enzyme activity of transgenic plants was higher than the control under all treatments. The largest gap in enzyme activity appeared in the cold treatment and the second-largest difference occurred under drought conditions (as shown in Figure 8B,C). In conclusion, overexpressing *FvMYB103* strongly improves a plant’s resistance to environmental stresses and plays a direct role in how plants manage drought and salt.

## 3. Discussion

As a form of abiotic stress, cold severely limits plants’ growth and development. A plant’s low temperature was categorized into two main types: chilling injury and freezing injury [31]. Plants activate complex internal adaptations to survive in response to low-temperature stress. Cold hardiness is achieved through the control of specific cold-responsive genes [32]. One of the largest and most functionally diverse TF families, MYB TFs regulate fundamental plant processes, including directing cell division, timing flowering, and organizing stress responses [33]. Within this family, the R2R3-MYB group is especially important for environmental resilience. Research has identified numerous R2R3-MYB TFs which help plants to resist disease [34,35], tolerate salt, and survive drought [36]. In the model plant *Arabidopsis thaliana*, proteins like AtMYB60, AtMYB96, and AtMYB61 are involved in regulating drought tolerance [37,38,39]. We demonstrate that *FvMYB103* can enhance cold tolerance in *Arabidopsis*. This result underscores the pivotal role of MYB TFs in adapting to physical stresses. The woodland strawberry (*F. vesca*) serves as an excellent model for studying these traits, possessing a short life cycle and stable genetic traits of characteristics [40]. *F. vesca* boasts abundant wild resources, whose genome was first published in 2011 [41] and, after multiple rounds of annotation and refinement, now constitutes sophisticated genome information [42,43]. China, for instance, possesses relatively rich germplasm resources of these wild species, which provide a reservoir to enhance cold tolerance [44]. A researcher found by analyzing three diploid strawberry species with different low-temperature treatments that wild strawberries typically succumb at temperatures between −5 °C and −10 °C [45].

Our study identified 106 R2R3-FvMYBs in the wild strawberry and compared these genes to those from *Arabidopsis thaliana* by constructing a phylogenetic tree. The analysis suggests that the R2R3-FvMYBs have diversified significantly. We then examined the conserved structures and found substantial variation in amino acid lengths. Functionally, about 75% of R2R3-FvMYBs fall within the 220–400 amino acid range, consistent with the typical structures of R2R3-MYB TFs. R2R3-MYB proteins within the same evolutionary subfamily usually perform similar roles [17,46]. FvMYB19 and FvMYB28, which cluster with the *Arabidopsis* S2 subfamily, may regulate the phenylpropanoid biosynthesis pathway [47]. FvMYB87 and FvMYB97, which cluster with the *Arabidopsis* S6 subfamily, likely participate in producing anthocyanins, which are plant pigments [48,49]. Variations in the amino acid count can directly influence other properties; the molecular weight of R2R3-FvMYB proteins is highly correlated with amino acids. Both stable and unstable proteins were identified among R2R3-FvMYBs. Stable proteins probably support core cellular functions, with their longevity allowing them to maintain steady conditions in the cell. Furthermore, all R2R3-FvMYB proteins showed hydrophilic characteristics which suggests that most of them are located in the cell’s nucleus [50,51].

Tandem duplication and segmental duplication are primary modes of gene duplication responsible for gene family expansion [52]. In *Fragaria vesca*, a substantial number of syntenic links involving R2R3-FvMYBs were identified across different chromosomes, indicating widespread segmental duplications within its genome [53,54]. R2R3-FvMYBs are not evenly spread across the chromosomes; we found several gene clusters resulting from tandem duplications on specific chromosomes. In contrast, the grape genome relied more heavily on segmental duplication for expansion [55].

Analysis of RNA-seq data from cold-treated strawberry stems and leaves identified a set of differentially expressed genes (DEGs), providing clues to their potential functions. Subsequently, we selected four R2R3-FvMYBs to analyze the expression patterns under cold treatment. The results indicated that at 4 °C treatment, *FvMYB94*, *FvMYB28*, and *FvMYB103* in leaves, and *FvMYB94*, *FvMYB28*, *FvMYB76*, and *FvMYB103* in stems, and at −4 °C treatment, *FvMYB28* and *FvMYB103* in leaves, and *FvMYB94*, *FvMYB28*, *FvMYB76*, and *FvMYB103* in stems, exhibited strong responses to cold. In this study, after overexpressing *FvMYB103* in *Arabidopsis*, no obvious phenotypic changes were observed at room temperature. However, under low-temperature treatment, the root length of the overexpression lines was significantly greater than that of the control. Related studies have shown that in cold environments, cold signals are perceived and transduced into cells, promoting a series of changes at the levels of gene expression, protein expression, and biochemistry [56,57].

In this study, under low-temperature stress, *Arabidopsis* plants overexpressing *FvMYB103* exhibited significantly higher activities of POD and SOD enzymes compared to wild-type plants. These findings indicate that *FvMYB103* acts as a positive regulator, enhancing the ability of *Arabidopsis* to withstand cold. We also assessed the performance of *FvMYB103*-overexpressing *Arabidopsis* under drought and salt stress conditions. POD and SOD are crucial antioxidant enzymes that scavenge reactive oxygen species and degrade hydrogen peroxide, thereby regulating the redox homeostasis in plants [58,59]. Yang found that *IlMYB306* increased the activities of POD and SOD, as well as free proline content, in transgenic tobacco under low-temperature treatment [51]. Research reported that *Arabidopsis* overexpressing *BcMYB111* showed increased levels of SOD and POD in Chinese cabbage, but the activities of malondialdehyde (MDA) and hydrogen peroxide (H_2_O_2_) decreased in the plants, which demonstrates a genuinely enhanced antioxidant capacity within plant cells. BcMYB111 can physically interact with BcCBF2 to activate the expression of *BcMYB111*, which enhances a plant’s resistance to low-temperature stress [60]. In summary, the study shows that the FvMYB103 protein can elevate key antioxidant enzyme activities and directly improve a plant’s resistance to environmental stress. However, the precise molecular mechanism behind the role of *FvMYB103* in cold tolerance in *F. vesca* is not yet defined and requires further investigation.

## 4. Materials and Methods

### 4.1. Plant Materials and Grow Stress Experiments

The *Fragaria vesca* ‘Hawaii’ seedlings (GDR, https://www.rosaceae.org, accessed on 10 May 2025, accession number: PRJNA60037) used in the experimental laboratory were provided by the research group of the School of Horticulture and Landscape Architecture of Northeast Agricultural University, and the seedlings were transplanted from the horticultural station of Northeast Agricultural University into a sterilized substrate (grass charcoal soil: vermiculite = 3:1) and placed in an artificial climate incubator. The incubation conditions were set as follows: light period 16 h light/8 h dark, light intensity 150 μmol·m^−2^·s^−1^, day/night temperature 22 ± 1 °C/18 ± 1 °C, and air relative humidity maintained at 60–70%. We prepared seventy-five-day-old *F. vesca* for analysis by incubating them at three distinct temperatures: room temperature (22 °C), 4 °C, and −4 °C for 8 h, respectively. Each experiment was performed with 3 biological replicates. Following this treatment, we collected samples of leaf and stem tissue for subsequent experimental.

Primers are designed by coding sequence region of *FvMYB103*, which is amplified via polymerase chain reaction (PCR). The resulting DNA fragment was ligated into a pCAMBIA1300-GFP vector (The detailed primer sequences used in this study are listed in Table S3). Transgenic plants were generated through Agrobacterium tumefaciens (GV3101)-mediated transformation of *Arabidopsis thaliana* Columbia ecotype (Col) [61]. We screened the treated *Arabidopsis* plants on a selective medium, which consisted of a 1/2 Murashige and Skoog (MS) mix containing 100 mg/L kanamycin. After 7 days of growth, the plants were transplanted into a culture pot containing nutrient soil and vermiculite (16 h light/8 h dark, light intensity 150 μmol·m^−2^·s^−1^, day/night temperature 22 ± 1 °C/18 ± 1 °C, and air relative humidity maintained at 60–70%), and after 28 days of growth, all plant leaves were collected and stored in an ultra-low temperature refrigerator, and the untreated leaves were used as controls to explore the changes in physiological indicators of plants caused by cold stress. After extracting genomic DNA from the surviving plants, we confirmed the successful transformation of *FvMYB103* by PCR analysis [62]. The confirmed transgenic seedlings grew for approximately seven days, then were transferred to pots containing a mixture of nutrient soil and vermiculite and grew under standard conditions for 40 days. Subsequently, wild-type and *FvMYB103*-overexpressing plants were subjected to abiotic stress tolerance assays. For the freezing stress test, we treated plants at −4 °C for ten hours, watered plants with a 200 mM NaCl solution for five days for salt stress test, and withheld water from plants for five days for drought stress test [63]. We documented the physical appearance of all plants both before and after each stress treatment. We also collected leaf samples from these plants that quantify the activity of peroxidase (POD) and superoxide dismutase (SOD) using the nitroblue tetrazolium (NBT) photoreduction method; each experiment was performed with 3 biological replicates [64,65].

### 4.2. Real-Time Fluorescenece Quantitative PCR Validation

We extracted total RNA from *Fragaria vesca* samples by using the FastPureCell/Tissue Total RNA Isolation Kit V2 (Vazyme, Nanjing, China). We then reverse transcribed the purified RNA into cDNA. We performed this step by following the instructions provided with the qRT-PCR kit (Vazyme, China). We performed quantitative PCR using a Blaze Taq™ SYBR^®^ Green qPCR Mix2.0 kit (Vazyme, China). Each reaction had a total volume of 20 μL. The reaction mixture contained 4 μL of 5× Premix, 2 μL of gene-specific primers, 2 μL of cDNA template, and 10 μL of ddH_2_O [66]. We ran the reactions on an ABI 7500 system. The thermal cycling parameters were as follows: an initial denaturation at 95 °C for 30 s, followed by 43 cycles of 95 °C for 5 s and 60 °C for 30 s [67]. We used the FaActin gene as an internal reference to normalize the data. We performed each experiment with three separate biological replicates. We analyzed the resulting data using the 2^−ΔΔCt^ method [68,69].

### 4.3. The Identification of R2R3-MYB Gene Family in Fragaria vesca

The genomic data of *F. vesca* from the Genome Database for Rosaceae were downloaded (GDR, https://www.rosaceae.org, accessed on 10 May 2025). We obtained the HMM profile for the MYB domain (PF00249) from the Pfam protein family database. An HMM was searched by using the TBtools-II software with this profile, with a set cutoff threshold value of E ≥ 0 for the search. All results that met this threshold were extracted and further analyzed for the corresponding protein sequences using the conserved domain (CD) search tool on the National Center for Biotechnology Information (NCBI) platform (https://www.ncbi.nlm.nih.gov/genome, accessed on 10 May 2025). Meanwhile, a separate search for the entire MYB domain name was conducted through a standard web browser. This double validation method was used to confirm the presence of MYB domains in the candidate sequences. We compared and consolidated the results from both search methods [70], then removed redundant gene sequences from this combined set. This process gave us our final list of candidate proteins. Each candidate protein belongs to the target gene family and contains at least one confirmed MYB domain.

The genomic data and annotation files for *Arabidopsis thaliana* were from their dedicated database (https://www.ncbi.nlm.nih.gov/Structure/cdd/wrpsb.cgi, accessed on 10 May 2025). We followed the same procedure used for *F. vesca* to obtain the protein sequences of the *Arabidopsis* R2R3-MYB gene family. Store the amino acid sequences of the R2R3-MYB gene family from *F. vesca* and the screened complete amino acid sequences of *Arabidopsis thaliana* in FASTA format. The amino acid sequences of strawberry and *Arabidopsis thaliana* were aligned multiple times by using MEGA11, and a phylogenetic tree from this alignment was constructed to use the neighbor-joining (NJ) method, with 1000 bootstrap replicates. The visualization of the phylogenetic tree was enhanced through iTOLv6 website (https://itol.embl.de/, accessed on 10 May 2025), and simultaneously classified R2R3-FvMYB gene family based on the tree [71].

Conserved motif analysis on R2R3-MYB gene family used MEME online tool (http://meme-suite.org/tools/meme, accessed 10 May 2025) with its default parameters. We specified the number of conserved motifs for the search. TBtools-II software was used to visualize the results of conserved motif analysis, and integrated diagrams were generated to show the phylogenetic relationships, conserved motifs, protein domains, and gene structures of R2R3-FvMYB gene family.

### 4.4. Chromosomal Localization and Study of Cis-Acting Elements

We downloaded the genome annotation file for *F. vesca* version 6.0 from the Genome Database for Rosaceae (GDR). The specific chromosomal locations of all R2R3-FvMYBs were determined by using TBtools software, then we renamed these genes in sequential order based on their positions along the chromosomes. Next, we performed a self-genome alignment for *F. vesca* genome. We analyzed the genome files using the Multiple Collinearity Scan toolkit (MCScanX). We ran this analysis with the default parameter settings, including an E-value cutoff of ≤1 × 10^−10^. We performed a visual analysis on the results files from MCScanX. This step allowed us to construct a Circos plot to visualize the genomic collinearity.

We analyzed the cis-acting regulatory elements in the promoter regions of R2R3-MYB gene family. First, we extracted 2000 base pair sequences located directly upstream of transcription start site (TSS) for each gene. We obtained these sequences from the cultivated strawberry genome annotation and gene sequence files. These promoter sequences were downloaded to the PlantCARE database website (http://bioinformatics.psb.ugent.be/webtools/plantcare/html/, accessed on 10 May 2025). This tool predicted the locations and types of cis-acting regulatory elements within each sequence. Finally, we used TBtools software to create a visual summary of the prediction results.

### 4.5. Collinearity Analysis of Genes

Process the files derived from the aforementioned chromosomal localization, compare the genomic data of woodland strawberry (*F*. *vesca*), *Arabidopsis thaliana*, and cultivated strawberry, identify homologous genes among these three species, and conduct synteny analysis across the three species.

### 4.6. Processing and Analysis of Transcriptome Data

Strawberry plants were treated as follows: Plants were first grown at 22 °C (room temperature) for 75 days, then divided into two treatment groups—one subjected to 4 °C treatment for 8 h, and the other to −4 °C treatment for 8 h—with three biological replicates for each treatment. Leaves and stems of plants from both the control group and treatment groups were collected for transcriptome sequencing to identify cold resistance-related R2R3-FvMYBs.

The sequenced data were subjected to quality assessment first, followed by aligning and mapping reads to the *F. vesca* reference genome using Hisat2. StringTie’s Cufflinks was used to quantify gene expression across different treatments, yielding the expression levels of R2R3-MYBs under each treatment, which were represented as Fragments Per Kilobase of transcript per Million mapped reads (FPKM) based on exon models. Comparative genomics analysis obtained related gene expression data and TBtools is used to convert mapped reads to transform log_2_ and plot as a heatmap. The red–blue color gradient in the heatmap indicates expression levels from high to low.

### 4.7. Transcriptome Data Processing and Expression Profiling

We treated strawberry plants at three different temperatures: room temperature (22 °C), 4 °C, and −4 °C. Each treatment lasted for six hours. After the treatment, we collected leaf and stem samples. The experiment was performed with three biological replicates. We conducted transcriptome sequencing on the collected samples. The goal of this sequencing was to identify cold resistance-related genes within R2R3-FvMYB gene family. The raw sequencing data were quality-assessed using FastQC (v0.11.9). Reads were then aligned to the strawberry (*F. vesca* L.) reference genome using Hisat2 (v2.2.1). Expression levels of R2R3-FvMYBs under different temperature treatments were quantified using Cufflinks within the StringTie package (v2.2.1) and are presented as Fragments Per Kilobase of transcript per Million mapped reads (FPKM). Gene expression data were obtained through genome alignment [72]. Data with FPKM > 1 were log_2_-transformed and subsequently visualized as a heatmap using TBtools. The color gradient from red to blue represents expression levels from high to low.

## 5. Conclusions

In summary, we identified 106 R2R3-FvMYB transcription factors and classified them into 25 subfamilies, localized in seven chromosomes of *F. vesca*, exhibiting chromosomal localization inhomogeneity and tandem replication events on specific chromosomes. Physicochemical property analysis indicated that the encoded R2R3-MYB proteins shared a conserved motif, functional domains, and structural organization. The grand average of hydropathicity (GRAVY) for each protein further showed that most R2R3-FvMYBs proteins are hydrophilic. The analysis of cis-acting elements provided functional predictions for R2R3-MYBs and laid the foundation for subsequent research. Transcriptome data analysis and qRT-PCR validation indicated that the expression of *FvMYB103* markedly increased in the roots and stems of *F. vesca* under treatments at both 4 °C and −4 °C. Obviously, overexpression of *FvMYB103* in *Arabidopsis* significantly enhanced cold tolerance, and the same situation occurred with salt stress (irrigation with 200 mM NaCl for 5 days) and drought stress (water withholding for 5 days). Meanwhile, we measured the activity of POD and SOD in *Arabidopsis thaliana* under four different treatments, all of which showed that transgenic *Arabidopsis thaliana* produced more POD and SOD in the face of abiotic stress. However, the research remains immature. We need to understand the full regulatory network of the molecular mechanisms of R2R3-FvMYB TFs during cold stress. Ultimately, it is necessary and logical to apply knowledge to improve cold tolerance in cultivated strawberry varieties.

## Data Availability

Data are contained within the article and Supplementary Materials.

## Supplementary Materials

The following supporting information can be downloaded at https://www.mdpi.com/article/10.3390/ijms27020771/s1.
