# Supplementary material for: Genome-Wide Identification of R2R3-MYB Gene Family in Strawberry (Fragaria vesca L.) and Functional Characterization of FvMYB103 in Cold Stress
Source: Int J Mol Sci. 2026 Jan 13;27(2):771. doi: 10.3390/ijms27020771 (PMC12840808; doi:10.3390/ijms27020771)
Supplement: Supplementary file 1 [file ijms-27-00771-s001.zip › ijms-4075708-supplementary.pdf]

Supplementary Materials

Supplementary Table S1.List of the gene structures and physicochemical properties of R2R3-MYB gene family in this study.

Sequence ID	Number of Amino Acid	Molecular Weight	Theoretical pI	Instability Index	Grand Average of Hydropathicity
FvesChr1G00282440.1	319	36116.90	6.64	67.80	-0.971
FvesChr1G00289180.1	270	30921.39	5.14	53.39	-0.840
FvesChr1G00289210.1	296	33075.97	6.15	51.28	-0.704
FvesChr1G00299370.1	309	33816.88	8.67	59.15	-0.617
FvesChr1G00301440.1	363	40642.43	7.63	50.39	-0.679
FvesChr1G00304760.1	349	38797.57	5.82	48.84	-0.496
FvesChr1G00306600.1	235	27534.05	8.85	46.45	-0.805
FvesChr1G00311760.1	315	35793.56	5.22	57.64	-0.651
FvesChr1G00316160.1	340	37839.29	7.18	49.84	-0.684
FvesChr1G00316880.1	312	34033.14	7.13	52.53	-0.773
FvesChr1G00319570.1	418	46951.81	8.11	48.64	-0.722
FvesChr2G00179850.1	266	29408.41	9.08	55.02	-0.539
FvesChr2G00184850.1	270	30074.76	8.31	56.52	-0.765
FvesChr2G00196170.1	639	71319.70	6.35	59.55	-1.115
FvesChr2G00197870.1	362	40641.94	9.54	62.81	-0.812
FvesChr2G00201290.1	655	74722.99	8.69	64.95	-0.972
FvesChr2G00205140.1	513	56209.46	5.61	71.62	-0.691
FvesChr2G00206080.1	272	30450.84	5.07	50.81	-0.672
FvesChr2G00217160.1	223	25813.05	8.83	53.70	-0.806
FvesChr2G00217200.1	228	26036.05	7.64	53.86	-0.928
FvesChr2G00217240.1	294	32987.97	6.34	46.39	-0.730
FvesChr2G00217250.1	282	32045.75	5.14	62.02	-0.669
FvesChr2G00217260.1	280	31021.82	6.34	41.17	-0.635

FvesChr2G00218680.1	420	46573.26	6.64	51.28	-0.607
FvesChr2G00220050.1	326	35107.70	9.43	54.34	-0.429
FvesChr2G00221430.1	246	27051.89	8.86	53.16	-0.720
FvesChr2G00223740.1	309	34060.82	5.41	39.10	-0.725
FvesChr2G00225430.1	357	41253.09	9.23	65.81	-0.894
FvesChr3G00068130.1	303	33350.21	5.95	50.83	-0.769
FvesChr3G00068140.1	294	32290.70	6.37	45.63	-0.487
FvesChr3G00072570.1	259	29444.70	5.35	54.51	-0.765
FvesChr3G00072690.1	366	42024.57	5.67	51.52	-0.780
FvesChr3G00083070.1	310	35224.47	5.90	57.68	-0.694
FvesChr3G00083900.1	339	37958.55	6.43	57.44	-0.642
FvesChr3G00083960.1	372	41439.58	8.56	55.14	-0.827
FvesChr3G00085470.1	377	42127.79	5.08	48.03	-0.775
FvesChr3G00103690.1	335	37348.33	5.05	55.78	-0.705
FvesChr3G00117790.1	464	52286.17	9.42	47.21	-0.588
FvesChr3G00118010.1	518	57813.79	7.77	53.35	-0.892
FvesChr3G00122110.1	326	36325.78	6.31	40.39	-0.704
FvesChr4G00131750.1	256	28718.01	5.06	53.08	-0.636
FvesChr4G00133610.1	264	30502.20	8.87	63.60	-0.805
FvesChr4G00140140.1	363	41073.85	6.01	53.60	-0.712
FvesChr4G00150640.1	320	34774.65	6.20	55.07	-0.347
FvesChr4G00155660.1	383	42796.71	5.37	55.12	-0.705
FvesChr4G00157290.1	340	38151.85	5.99	50.21	-0.790
FvesChr4G00164860.1	283	32085.98	5.62	73.03	-0.649
FvesChr4G00169060.1	416	45685.25	6.81	47.05	-0.562
FvesChr4G00169270.1	410	45495.22	6.61	65.07	-0.703
FvesChr4G00169310.1	333	37356.19	7.66	46.17	-0.683

FvesChr4G00169800.1	479	54000.25	6.45	62.24	-0.822
FvesChr4G00170480.1	255	29224.91	8.60	47.26	-0.878
FvesChr4G00176370.1	291	31118.68	6.06	50.17	-0.545
FvesChr5G00229600.1	279	31253.96	4.98	49.30	-0.626
FvesChr5G00231170.1	452	50847.73	7.20	53.36	-0.678
FvesChr5G00232680.1	353	39997.76	5.69	56.92	-0.584
FvesChr5G00235380.1	256	29297.30	6.00	47.00	-1.006
FvesChr5G00236270.1	377	42382.98	5.76	50.41	-0.634
FvesChr5G00242470.1	223	25348.54	8.23	37.23	-0.802
FvesChr5G00243020.1	284	32987.63	5.82	60.07	-0.857
FvesChr5G00243960.1	303	32635.33	7.08	58.19	-0.625
FvesChr5G00246290.1	289	32130.21	9.02	44.99	-0.579
FvesChr5G00246300.1	317	35334.80	8.22	46.56	-0.573
FvesChr5G00246650.1	358	39848.05	6.72	36.96	-0.816
FvesChr5G00246920.1	285	32311.35	8.81	47.31	-0.780
FvesChr5G00249360.1	188	21409.53	8.99	42.71	-0.699
FvesChr5G00249370.1	191	21606.39	8.46	49.47	-0.804
FvesChr5G00250350.1	277	32512.17	8.86	61.74	-1.016
FvesChr5G00254360.1	311	34255.04	6.82	52.10	-0.462
FvesChr5G00257700.1	287	32644.58	6.68	39.59	-0.922
FvesChr5G00263660.1	342	37966.87	8.32	56.13	-0.818
FvesChr5G00272610.1	127	15016.27	10.19	52.06	-1.035
FvesChr5G00275790.1	322	36208.52	5.95	53.28	-0.625
FvesChr6G00002020.1	425	47363.21	5.05	62.69	-0.879
FvesChr6G00010180.1	441	49138.29	7.28	56.23	-0.743
FvesChr6G00013440.1	248	28186.97	6.72	51.85	-0.523
FvesChr6G00014130.1	313	35430.12	6.73	50.79	-0.883

FvesChr6G00014790.1	386	42508.72	7.59	43.74	-0.645
FvesChr6G00016210.1	357	41297.17	9.38	61.38	-0.896
FvesChr6G00019710.1	317	35384.50	7.56	49.49	-0.688
FvesChr6G00019930.1	229	26615.51	5.15	59.20	-0.858
FvesChr6G00028370.1	403	45379.42	6.68	69.44	-0.826
FvesChr6G00042110.1	249	28632.58	5.04	50.04	-0.951
FvesChr6G00043350.1	383	42485.77	6.12	60.89	-0.355
FvesChr6G00044290.1	228	26108.23	8.72	59.57	-0.996
FvesChr6G00046470.1	488	53312.30	5.66	66.98	-0.644
FvesChr6G00049530.1	212	24010.03	6.14	49.50	-0.760
FvesChr6G00056270.1	266	30401.82	4.99	43.33	-0.691
FvesChr6G00056280.1	289	33031.15	5.43	68.23	-0.932
FvesChr6G00056330.1	372	41324.22	5.85	54.74	-0.518
FvesChr6G00057580.1	339	38450.65	9.18	62.91	-0.802
FvesChr6G00058740.1	366	41485.59	6.54	47.36	-0.674
FvesChr6G00060780.1	371	40672.03	6.18	50.65	-0.675
FvesChr6G00061380.1	360	39442.95	6.16	45.39	-0.580
FvesChr6G00062610.1	414	45397.19	6.46	59.24	-0.362
FvesChr6G00063910.1	207	23207.98	7.73	49.49	-0.820
FvesChr6G00063990.1	549	62778.73	8.33	60.30	-0.997
FvesChr7G00321150.1	334	37648.00	6.18	64.26	-0.716
FvesChr7G00326380.1	562	61175.23	5.14	56.22	-0.585
FvesChr7G00328870.1	223	24793.55	5.79	55.08	-0.698
FvesChr7G00333040.1	325	36752.40	4.97	57.96	-0.662
FvesChr7G00338200.1	340	38255.97	6.21	50.30	-0.661
FvesChr7G00341810.1	284	32319.44	9.18	52.79	-0.919
FvesChr7G00349190.1	398	43822.40	6.77	41.10	-0.563

FvesChr7G00349490.1	304	34568.38	6.46	65.09	-0.831
FvesChr7G00356160.1	290	33002.69	6.70	54.04	-0.880

Supplementary Table S2.List of Gene ID and Gene Name in this study.

Gene ID	Gene Name
FvesChr1G00282440.1	FvMYB1
FvesChr1G00289180.1	FvMYB2
FvesChr1G00289210.1	FvMYB3
FvesChr1G00299370.1	FvMYB4
FvesChr1G00301440.1	FvMYB5
FvesChr1G00304760.1	FvMYB6
FvesChr1G00306600.1	FvMYB7
FvesChr1G00311760.1	FvMYB8
FvesChr1G00316160.1	FvMYB9
FvesChr1G00316880.1	FvMYB10
FvesChr1G00319570.1	FvMYB11
FvesChr2G00179850.1	FvMYB12
FvesChr2G00184850.1	FvMYB13
FvesChr2G00196170.1	FvMYB14
FvesChr2G00197870.1	FvMYB15
FvesChr2G00201290.1	FvMYB16
FvesChr2G00205140.1	FvMYB17
FvesChr2G00206080.1	FvMYB18
FvesChr2G00217160.1	FvMYB19
FvesChr2G00217200.1	FvMYB20
FvesChr2G00217240.1	FvMYB21

FvesChr2G00217250.1	FvMYB22
FvesChr2G00217260.1	FvMYB23
FvesChr2G00218680.1	FvMYB24
FvesChr2G00220050.1	FvMYB25
FvesChr2G00221430.1	FvMYB26
FvesChr2G00223740.1	FvMYB27
FvesChr2G00225430.1	FvMYB28
FvesChr3G00068130.1	FvMYB29
FvesChr3G00068140.1	FvMYB30
FvesChr3G00072570.1	FvMYB31
FvesChr3G00072690.1	FvMYB32
FvesChr3G00083070.1	FvMYB33
FvesChr3G00083900.1	FvMYB34
FvesChr3G00083960.1	FvMYB35
FvesChr3G00085470.1	FvMYB36
FvesChr3G00103690.1	FvMYB37
FvesChr3G00117790.1	FvMYB38
FvesChr3G00118010.1	FvMYB39
FvesChr3G00122110.1	FvMYB40
FvesChr4G00131750.1	FvMYB41
FvesChr4G00133610.1	FvMYB42
FvesChr4G00140140.1	FvMYB43
FvesChr4G00150640.1	FvMYB44
FvesChr4G00155660.1	FvMYB45
FvesChr4G00157290.1	FvMYB46
FvesChr4G00164860.1	FvMYB47
FvesChr4G00169060.1	FvMYB48

FvesChr4G00169270.1	FvMYB49
FvesChr4G00169310.1	FvMYB50
FvesChr4G00169800.1	FvMYB51
FvesChr4G00170480.1	FvMYB52
FvesChr4G00176370.1	FvMYB53
FvesChr5G00229600.1	FvMYB54
FvesChr5G00231170.1	FvMYB55
FvesChr5G00232680.1	FvMYB56
FvesChr5G00235380.1	FvMYB57
FvesChr5G00236270.1	FvMYB58
FvesChr5G00242470.1	FvMYB59
FvesChr5G00243020.1	FvMYB60
FvesChr5G00243960.1	FvMYB61
FvesChr5G00246290.1	FvMYB62
FvesChr5G00246300.1	FvMYB63
FvesChr5G00246650.1	FvMYB64
FvesChr5G00246920.1	FvMYB65
FvesChr5G00249360.1	FvMYB66
FvesChr5G00249370.1	FvMYB67
FvesChr5G00250350.1	FvMYB68
FvesChr5G00254360.1	FvMYB69
FvesChr5G00257700.1	FvMYB70
FvesChr5G00263660.1	FvMYB71
FvesChr5G00272610.1	FvMYB72
FvesChr5G00275790.1	FvMYB73
FvesChr6G00002020.1	FvMYB74
FvesChr6G00010180.1	FvMYB75

FvesChr6G00013440.1	FvMYB76
FvesChr6G00014130.1	FvMYB77
FvesChr6G00014790.1	FvMYB78
FvesChr6G00016210.1	FvMYB79
FvesChr6G00019710.1	FvMYB80
FvesChr6G00019930.1	FvMYB81
FvesChr6G00028370.1	FvMYB82
FvesChr6G00042110.1	FvMYB83
FvesChr6G00043350.1	FvMYB84
FvesChr6G00044290.1	FvMYB85
FvesChr6G00046470.1	FvMYB86
FvesChr6G00049530.1	FvMYB87
FvesChr6G00056270.1	FvMYB88
FvesChr6G00056280.1	FvMYB89
FvesChr6G00056330.1	FvMYB90
FvesChr6G00057580.1	FvMYB91
FvesChr6G00058740.1	FvMYB92
FvesChr6G00060780.1	FvMYB93
FvesChr6G00061380.1	FvMYB94
FvesChr6G00062610.1	FvMYB95
FvesChr6G00063910.1	FvMYB96
FvesChr6G00063990.1	FvMYB97
FvesChr7G00321150.1	FvMYB98
FvesChr7G00326380.1	FvMYB99
FvesChr7G00328870.1	FvMYB100
FvesChr7G00333040.1	FvMYB101
FvesChr7G00338200.1	FvMYB102

FvesChr7G00341810.1	FvMYB103
FvesChr7G00349190.1	FvMYB104
FvesChr7G00349490.1	FvMYB105
FvesChr7G00356160.1	FvMYB106

Supplementary Table S3.List of primers in this study.

Primer	Sequence(5'-3')	Purpose
FvMYB103-F	ATGGGAAGGGCTCCTTGTTG	Full-length cDNA of FvMYB103
FvMYB103-R	TTAGATCAGCAGCGACTCAGC	Full-length cDNA of FvMYB103
FvMYB103-qF	GGGTCGATGTCGTCGGATTT	qPCR
FvMYB103-qR	TTAGCTTGCAGGCAGCATTC	qPCR
FvMYB94-qF	CCCAGTTAAGCTCAGCCACA	qPCR
FvMYB94-qR	AGGGCATTTACTCCAAGGGC	qPCR
FvMYB28-qF	AGCTGTGCAGCCTATTGAGG	qPCR
FvMYB28-qR	GAAAGCCAGGGAGGAAGCAT	qPCR
FvMYB76-qF	GTTGCCAATTGGGCGTTAGG	qPCR
FvMYB76-qR	CTGCAAAGTGGTAAACGGGC	qPCR
FvActin-qF	GGGCCAGAAAGATGCTTATGTCGG	qPCR
FvActin-qR	GGGCAACACGAAGCTCATTGTAGAAG	qPCR
